# Geographical Prevalence of Polycystic Ovary Syndrome as Determined by Region and Race/Ethnicity

**DOI:** 10.3390/ijerph15112589

**Published:** 2018-11-20

**Authors:** Wendy M. Wolf, Rachel A. Wattick, Olivia N. Kinkade, Melissa D. Olfert

**Affiliations:** 1 Physician Assistant and Registered Dietitian at Apex Family Medicine, Denver, CO 80209, USA; wendywolf02@gmail.com; 2 Department of Human Nutrition and Foods, West Virginia University, Morgantown, WV 26506, USA; rawattick@mix.wvu.edu (R.A.W.); onk0001@mix.wvu.edu (O.N.K.)

**Keywords:** polycystic ovary syndrome, prevalence, diagnostic criteria, race, ethnicity, regions

## Abstract

Polycystic ovarian syndrome (PCOS) is thought to be the most common endocrine disorder found in women. Common symptoms include irregular menstrual cycle, polycystic ovaries, and hirsutism, as well as an increased risk for a multitude of conditions, including insulin resistance, dyslipidemia and infertility. The prevalence of polycystic ovarian syndrome is generally thought to be between 3% and 10% but it is widely unknown for specific subpopulations based on geographical location and race/ethnicity. Based on the high degree of variability and inconsistencies between the different diagnostic criteria, there is a unique challenge that exists when determining the prevalence of this syndrome. There are a large percentage of individuals that remain undiagnosed even after visiting multiple health care providers. Most studies conducted across the world are limited by small sample size, selection bias, and lack of comparability across studies. There have been very few studies that have examined the prevalence of polycystic ovary syndrome across the United States. Based on the National Institutes of Health (NIH)’s diagnostic criteria, there is a similar prevalence of PCOS documented across the United States, the United Kingdom, Spain, Greece, Australia, and Mexico. Other studies have shown some differences between geographical location and race. The existing data is not conclusive enough to determine whether or not there is any significant differences in the prevalence of PCOS across geographical location, racial or ethnic groups. This review will seek to determine the prevalence of polycystic ovarian syndrome based on geographical location and race/ethnicity.

## 1. Introduction

Polycystic ovarian syndrome (PCOS) is thought to be the most common endocrine disorder found in women [1,2,3]. PCOS impacts women of all races and ethnicities who are of reproductive age. In unspecified populations the prevalence of PCOS has a reported incidence rate of 3–10% [4,5]. PCOS is a syndrome that is seen only in women and is most often characterized by an imbalance of the sex hormones [1]. Common symptoms include irregular menstrual cycle, polycystic ovaries, and hirsutism [2]. Features of the syndrome may also include infertility, insulin-resistance, impaired glucose tolerance (Type 2 Diabetes), and dyslipidemia, due to increased risk factors [1,6]. The etiology of PCOS is not completely understood and there is no known cause, although a genetic component and diet/lifestyle factors, such as insulin resistance and obesity have been identified [7]. Given the impact of insulin resistance and obesity on the development of PCOS, this could be related to the difference in prevalence in across different races/ethnicities with higher incidence of obesity and diabetes. Due to the heterogeneous and multifactorial nature of PCOS symptoms there is a lack of a clear, agreed upon definition and diagnostic criteria [1,8].

PCOS has a very high percentage of individuals who remain undiagnosed when visiting their doctor, estimated to be as high as 75% [7,9]. This is likely due to variability of patient presentation and lack of provider knowledge. The benefit of capturing more of these patients would be linkage to care, increased screening for comorbidities, and overall improvement in patient care. Giving a patient the diagnosis of PCOS makes the patient aware of possible fertility concerns, dysfunctional bleeding, endometrial cancer, obesity, diabetes, dyslipidemia, hypertension, and theoretical increased risk of cardiovascular disease [7]. Since PCOS could be genetic, it may bring awareness to family members and future children. Given that insulin resistance is heavily associated with PCOS, these individuals require increased screening and will likely have better long-term outcomes with early lifestyle interventions, as well as insulin sensitizing medication, such as metformin when indicated. In addition, screening for hyperlipidemia could lead to earlier lifestyle/medical intervention could likely help reduce one’s cardiovascular outcomes.

In the current literature, there is a large disconnect between the prevalence of PCOS, geographical regions and race/ethnic factors. There are few studies that have examined specific subpopulations. These studies are commonly limited by small sample sizes, selection bias, and are not comparable with other studies’ findings, due to inconsistencies with the diagnostic criteria for PCOS. In order to fully understand the complexity and occurrence of PCOS, the prevalence needs to be assessed in the subpopulations. It is important for the field to reach the level of comprehension with PCOS to the extent that diabetes and metabolic syndrome established to improve the quality of life for these women [10,11]. This review will seek to determine the prevalence of polycystic ovarian syndrome based on geographical location and race/ethnicity. This will help to determine how much is understood regarding the risk and diagnosis of PCOS in specific regions of the world.

## 2. Review

### 2.1. Understanding the Prevalence of PCOS

In order to begin to understand what is currently known about the prevalence in subgroup populations of PCOS the complexity and issues of the current diagnostic criteria must be understood. There are three different sets of diagnostic criteria that used in the field (See Table 1) which have been set by National Institutes of Health (NIH)’s international conference on PCOS in 1990, the European Society of Human Reproduction and Embryology and the American Society for Reproductive Medicine (ESHRE/ASRM) in 2003 (referred to as the Rotterdam criteria), and the Androgen Excess Society and PCOS Society (AE-PCOS) in 2006 [6,7,12,13]. Each set of criteria has slightly different clinical, biological, and image-based findings to determine the presence or absence of PCOS [1]. The 1990 NIH Criteria suggest that a patient has PCOS if she displays symptoms of oligoovulation and androgen excess (clinical or biochemical) [6]. The Rotterdam 2003 Criteria was developed in response to a need for broader diagnostic criteria [1]. In order to be diagnosed with PCOS under the Rotterdam criteria (ROT) the individual must exhibit symptoms in two out of three categories, which include oligo/anovulation, hyperandrogenism, and the presence of polycystic ovaries [6]. In 2006 the AE-PCOS published criteria which placed an emphasis on hyperandrogenism, with clinical or biochemical evidence of hyperandrogenism being required for diagnosis [6,12]. The most recent input regarding the diagnostic criteria came from NIH Evidence-based Methodology Workshop Panel on Polycystic Ovary Syndrome in 2012. The experts proposed two major suggestions involving the way we diagnosis and refer to PCOS. The first suggestion was to rename the disorder completely. This would allow the name to better reflect the complexity of physiologic factors involved in this syndrome, such as metabolic, hypothalamic, pituitary, ovarian, and adrenal influences which contribute to the reproductive implications. Although a name change was recommended, no specific name was proposed. The second major change was to maintain the broad inclusive diagnostic criteria of Rotterdam 2003 (which includes NIH 1990 and AE-PCOS Society 2006 criteria), while specifically identifying subphenotypes. The proposed phenotypes included: (1) Androgen Excess + Ovulatory Dysfunction, (2) Androgen Excess + Polycystic Ovarian Morphology, (3) Ovulatory Dysfunction + Polycystic Ovarian Morphology, (4) Androgen Excess + Ovulatory Dysfunction + Polycystic Ovarian Morphology [14]. Each set of diagnostic criteria have specific exclusion criteria that excludes anyone with an underlying pathological condition that would explain hyperandrogenism or menstrual dysfunction, such as congenital adrenal hyperplasia, thyroid dysfunction, hyperprolactinemia [6,15]. Most recently, the 2018 International Guidelines for PCOS endorsed the Rotterdam criteria with a few caveats. An ultrasound is not needed for diagnosis if the patient has irregular menstrual cycles and hyperandrogenism is present, but it is still recommended for phenotyping. Adolescents should have more stringent guidelines and require hyperadrogenism and ovulatory dysfunction and be at least two years post menacrche. Ultrasound is not recommended for adolescents [15]. The diagnostic criteria are constantly evolving and is considered to be one of the most debated topics in the field of endocrinology making the prevalence of PCOS difficult to determine with consistency [12]. It has been previously stated that because PCOS is a clinical syndrome, there is no criteria that is fully sufficient for diagnosis [16].

### 2.2. Effect of the Diagnostic Criteria on Prevalence

Changes in the diagnostic criteria greatly affect the prevalence of PCOS. Prevalence rates have been reported as low as 1.6% using a combination of all three criteria [1] and as high as 18% [2] in similar Caucasian populations using the Rotterdam criteria [3,18]. A statistical report by Futterweit, estimated that 50–75% of women with PCOS are unaware that they even have this syndrome [9]. A retrospective cohort study by Amato et al. assessed a group of 204 age-matched women who were suspected to have PCOS to determine the difference in prevalence based on the diagnostic criteria [12]. This study found that the prevalence of PCOS in the identified population to be 51% according to NIH, 83% with ROT, 70.6% with AE-PCOS, and only 49% to fit the PCOS diagnosis under all three categorical descriptions [12]. These findings all showed a difference in the prevalence, as well as the frequency and severity of symptoms. A cohort study by Broekmans et al. analyzed a large anovulation-screening database. All cases were assessed under the Rotterdam criteria and then redefined and diagnosed under the NIH criteria to determine the prevalence of PCOS according to the two different definitions [8]. When the subjects were diagnosed according to the Rotterdam criteria there was a 1.5 times larger group that was diagnosed than when the same subjects were diagnosed using the NIH criteria. Under the Rotterdam criteria there was a greater frequency of obesity, insulin sensitivity, and the diagnosis of PCOS itself [8]. This study used appropriate groups and used the same subjects to assess an accurate depiction of the differences that can occur between the criteria. Another study found that the Rotterdam and AE-PCOS prevalence estimates were nearly twice that of the NIH criteria when classified on the same participants [2]. The lack of consistency and clarity between the diagnosis criteria affects the comparability and the standardization of all clinical treatments and research findings dealing with PCOS. A study in 2012 done by Yildiz et al. found the diagnostic criteria to greatly impact the prevalence [19]. This study looked at 392 volunteers who were all female, largely Caucasian employees of a government-based instituted located in Turkey between the ages of 18–45. Individuals were excluded if they were post-menopausal, on any medication (including oral contraception), history of hysterectomy or bilateral oophorectomy or if they were pregnant. There was an interview-based medical form to collect medical history and menstrual data, physical exam for anthropometric data, modified Ferriman–Gallwey (mF–G) method for hirsutism by a single physician (and re-examined by another physician if score equal to or greater than 3), blood work for hormone, cholesterol and biochemical analysis, and vaginal ultrasound. This study found that the prevalence was lowest with the NIH criteria at 6.1%, followed by 15.3% using the AE-PCOS criteria at 15.3%, and then highest with the Rotterdam criteria at 19.9%. They assessed the phenotypic presentations and the most common subphenotype was hyperandrogenism with polycystic ovaries. They also assessed prevalence compared to obese women and non-obese (BMI < 30 kg/m^2^) women. The prevalence of PCOS in obese (BMI ≥ 30 kg/m^2^) women according to NIH, Rotterdam and AE-PCOS Society criteria were 15%, 30%, and 18.8% respectively. This was significantly higher than the corresponding data in non-obese women—5.1%, 14.5% and 18.8%, respectively [20]. This was a relatively small study that found similar prevalence rates to the majority of studies done previously when using the NIH criteria, but they did find higher rates with the Rotterdam and AE-PCOS studies than prior studies. This variation could be because this study was lacking adequate power to fully assess prevalence (needed 400 subjects and they had 392). A comparative 2018 study done by Skiba et al. sought to understand the variances between all three diagnostic criteria [20]. Results showed that that were few differences between both NIH criteria and AE-PCOS criteria comparisons, as well as Rotterdam and AE-PCOS comparisons, but evaluations between NIH criteria and Rotterdam criteria showed significant disparities. This is thought to be due to the differences in assessment of ovarian morphology (ovaries found to have more than 12 follicles), the inclusion of which raises prevalence rates significantly [20]. This is why some recent studies have also shed light on issues concerning over-diagnosis. Ovarian cysts are a symptom that can be entirely unrelated to PCOS, and that are sometimes misdiagnosed as polycystic ovaries. As such, this confusion may sometimes skew prevalence rates significantly, as ovarian cysts alone should not be seen as a symptom of PCOS. Copp et al. speculated that over-diagnosis could also be due to the inclusion of more phenotypes in diagnostic criteria, as well as the recent inclusion of non-hyperandrogenic phenotypes under the Rotterdam criteria [21]. Non-hyperandrogenic women were found to have fewer long-term cofactor association with PCOS, and in some cases, were misdiagnosed with PCOS entirely, as menstrual irregularity and polycystic ovaries can be associated with other, unrelated conditions [21]. It is important to continue pushing diagnosis criteria to be as effective as possible, as the misdiagnosis of PCOS accomplishes nothing more than skewing prevalence rates and potentially preventing women from getting the accurate, appropriate care they need.

Another challenge in diagnosing PCOS are the evaluation methods used. Although there are guidelines that should be universally used, not all clinicians use the suggested criteria. The definition of PCOS follows strict criteria for diagnosis [22], as does defining hirsutism [23] Measuring hyperandrogenism is difficult due to some tools lacking sensitivity. These additional challenges add to the complexity of diagnosing PCOS.

There is a limited amount of literature on previous research regarding the prevalence of PCOS dependent on a geographical location, specific race or ethnicity, or how the prevalence of PCOS is related to the occurrence of additional health disparities. Previous studies are commonly limited by a small sample size, fewer than 400, conducted at only one facility, and are not fully comparable due to the lack of consistency in the use of diagnostic criteria [1]. It has been stated that there is a significant difference seen in the symptoms presented across geographical locations and between differing race/ethnic group [24] While current data does remain limited, a number of recent studies continue to show highly significant differences in prevalence of PCOS and its symptoms and co-factors, encouraging the need for increased exploration across all of these domains [25,26,27].

### 2.3. Prevalence of PCOS across the US

Limited research has been documented in the United States assessing at the different geographical trends. To our knowledge, Okoroh et al. [1] is the only study to assess and compare regional prevalence of PCOS and its various phenotypes across the United States (US) and the first to use all available criteria to estimate the prevalence in the US (see Table 2). This is also believed to be the one of the largest prevalence studies done on a geographically diverse population within the United States. This research showed a higher prevalence of PCOS concentrated in the southern US than anywhere else in the US, at 47.5%. This study showed that following the South, in order of decreasing prevalence, was the North Central (23.0%), West (18.7%), and then the North East with the lowest prevalence 10.3%) [1]. This was a large-scale study that analyzed a commercial database containing claim reports that were collected from 2003 to 2008 looking at over 12 million privately insured women aged 18–45 from geographically diverse states. Only 1.6% of women met at least one diagnostic criteria for PCOS [1]. This prevalence may be a low estimate, since this a retrospective study only had access to medical charts previously completed and did not see the patients directly for an extensive clinical exam. There is also the possibility of the information on the charts being improperly coded leading to missed diagnosis. Since it is not uncommon for PCOS to go undiagnosed, it is extremely plausible that this article underestimates the prevalence of PCOS significantly.

### 2.4. Prevalence of PCOS in Caucasians across the World

Various studies have assessed Caucasians in the US, Spain, and Australia to determine whether or not Caucasian populations in other countries showed similar prevalence rates across the world.

A previous study, by Asuncion et al. [28] prospectively estimated the prevalence of PCOS using a design similar to Knochenhauer et al. [4] that selected women in an unbiased manner by using data from 154 consecutive Caucasian blood donors at a hospital in Madrid, Spain. Using the NIH diagnostic criteria, the study found an incidence rate for PCOS of 6.5% [28]. One limitation of this study was that it was a small study and although the selection was not biased, it was not completely randomized and is unlikely to be representative of the population in that area.

A large retrospective birth cohort was designed by March et al. to create a representative estimate of the prevalence of PCOS in those born in Adelaide, Australia [2] (see Table 3). Seven hundred and twenty-eight subjects were assessed that were all born at a single maternity hospital and could be located, interviewed, and clinically examined. This study took into consideration the lack of consistency between the diagnostic criteria and assessed the patients according to each criterion to determine prevalence rates specific to the criteria. The study determined a prevalence of 8.7 ± 2.0%, 11.9 ± 2.4%, 10.2 ± 2.2% according to NIH, Rotterdam, and the AES criteria respectively. These numbers increased to 17.8 ± 2.8% under the Rotterdam criteria and 12.0 ± 2.4% according the AES criteria when imputed data was included for those women who did not have an ultrasound [2]. The main strengths of this study were that it is the largest and only community-based study that looked at the prevalence of PCOS in a nearly homogeneous Caucasian population. These incidence rates seem higher than those determined in the United States, however this population was primarily Caucasian. It is said to be comparable to the US [28] in terms of obesity rates and waist circumference, but the rates of Australians were still lower than in the Americans [29,30,31,32] Some studies have looked at Australian indigenous women and found very different results that are discussed below [33]. Across all studies conducted on Caucasian women in comparison with women of different ethnic backgrounds, Caucasian women did seem to see generally lower rates of prevalence of PCOS and its symptoms and co-factors, however multiple studies have pointed out that a cohort that continually sees lower rates of prevalence is South Asian women, with both Mani et al. and Ding et al. putting Southern/Southeastern Asian women in the categories for rates of lowest prevalence [34,35].

### 2.5. The Prevalence of PCOS across Different Races/Ethnicities

Overwhelming evidence has and continues to suggest that the prevalence of PCOS may vary between different races and ethnicities [25,26,36]. The following studies assessed the prevalence of PCOS using looking at specific race/ethnicities in a single geographical area.

#### 2.5.1. Asian

Due to the wide-variety of ethnic groups of Asians it is expected that the variance or symptoms between individuals’ different ethnicities will vary and this has been documented across multiple studies. A community-based, cross-sectional study assessed a random sample that was representative of the community of over 3000 women between the ages of 15 and 39. Kumarapeli, et al. found a prevalence rate of 6.3% (95% CI: 5.9–6.8%) based on the Rotterdam criteria [37]. The study used a survey to first narrow down the probable cases and controls and then performed a clinical examination to further reduce probable cases and then used ultrasound tests to confirm the identified PCOS cases. Over 90 percent of women self-reported symptoms of oligo/amenorrhea and/or hirsutism were confirmed to have PCOS according to Rotterdam criteria [37]. It is important to note, however, that when compared with Caucasian groups, Asian women did still tend to be less hirsute [36]. This method showed evidence that a simple questionnaire-based survey may be an accessible and simple tool that could be used for PCOS screening in South Asia and even other areas of the world. It must be considered, though, that while employing surveys may be a more initially straightforward, practical way to assess these populations, chances for mis-, under-, or over-diagnosis could be increased by shifting focus from a more well-rounded, medical, and clinical diagnosis process. In this study only 0.65% of those with PCOS had been previously diagnosed. It is suspected that there would be an increased prevalence of PCOS in Sri Lankan women when compared with Caucasians, due to the known link between type 2 Diabetes Mellitus and the high prevalence of diabetes in Sri Lanka [35,38]. Due to differing diagnostic criteria used in similar studies in the US it is difficult to compare the results directly to determine the similarity. These findings were consistent with those documented in Southern Europe [36]. It has been reported that the prevalence of PCOS is considered to be higher in South Asians than in Caucasians residing in the United Kingdom, but prevalence is not necessarily higher in non-United Kingdom Caucasians [33,34,35,39]. Fifty-two percent of Asian women who reside in the Indian subcontinent present with polycystic ovaries, which is considered to the highest reported prevalence [40]. The estimated prevalence of women with polycystic ovaries in the US is approximately 21% [41]. Although Japan has lower rates of obesity and hirsutism, the Japanese still have comparable rates of androgen excess and insulin resistance to the US and Italy [27]. No known studies have been published describing the prevalence of PCOS in Japan or Italy in order to compare the populations further. In a 2017 review study done by Ding et al. contrasting four ethnic groups (Chinese, Caucasian, Middle Eastern, and African American), Chinese women were actually found to have the lowest prevalence rates in comparison with the other three groups, with a Rotterdam criterion of 5.6% [34]. An observational study done by Chen et al. in 2008 [42], looked at an unselected group of women of reproductive age in Southern China at the time of their annual physical. Diagnostic criteria adhered to the 1990 NIH guidelines. They demonstrated a 2.2% prevalence, which was 20 of the 915 subjects that met diagnostic criteria for PCOS. This is a lower prevalence than most studies but that could be related the fact that this was an unselected population and did not seek volunteers that could led to a selection bias or possibly higher rates of individuals who remain undiagnosed. This could also signify that Chinese women have a lower prevalence.

#### 2.5.2. Hispanic

In a study by Moran [43] a prevalence of 6.0% (95% CI: 1.9–10.1%), as diagnosed by the NIH criteria, or 6.6% (95% CI: 2.3–10.9%) under the Rotterdam criteria was found in a homogenous group of Mexican women residing in Mexico City. This study could have been limited by a small sample size of 150 women who all joined the study voluntarily. Strengths of this study include the use of two diagnostic criteria sets and the fact that they were all assessed clinically, biochemically and by a pelvic ultrasound [43].

According to Goodarzi et al. [44] a significantly higher PCOS prevalence has been documented in Mexican-American women living in Los Angeles that is approaching 13% [5,43,44]. This study prevalence could be impacted by a confounding factor from bias stemming from their selection of individuals who all had a family history of coronary artery disease. There was no clinical evaluation with the subjects because this study relied solely on self-reported data via a questionnaire, which could significantly skew their results. Unlike most studies this study failed to remove patients with hyperandrogenism connected with a related disorder. Furthermore, when compared to both non-Hispanic White women, Hispanic women are also reported to have a “significantly higher prevalence” of hirsutism (93.8% vs. 86.8%), abnormal free androgen index (75.8% vs. 56.5%), abnormal homeostasis model assessment (52.3% vs. 38.4%), and hyperglycemia (14.8% vs. 6.5%). Interestingly, when contrasted with non-Hispanic black women, a cohort that normally has higher rates of prevalence than almost any other ethnic group, Hispanic women saw higher rates of metabolic syndrome and hypertriglyceridemia [45].

The differences between studies focused on Mexican women could imply that there is a difference in lifestyle from those residing in Mexico vs. United States. If the experimental designs were similar in methodology and better controlled, then this assumption could be more stated with more confidence. It is plausible that there is a higher prevalence of PCOS in Mexican-Americans, because in a study comparing Caucasian women and Mexican-American women with PCOS found that they have been found to have a higher age-specific prevalence of insulin resistance and a higher body mass index (BMI) when compared to non-Hispanic White people. The study used a smaller sample size of 83 participants but consistently observed significantly higher mean values for BMI, fasting insulin, and homeostasis model assessment (HOMA) in Mexican-American women compared to the Caucasian women [5]. One of the most prominent features of PCOS is insulin resistance, which is found in 50–70% of individuals with PCOS [44].

#### 2.5.3. African American

Despite the correlation between a higher prevalence of PCOS and a higher Black population in the Southern US, Knonchenhauer et al. [4] found that there are no significant racial differences between Caucasian and Black people living in Alabama with a prevalence of 4.7% and 3.4%, respectively [4]. This study had a sample size of 369 women in the Southeastern United States that were between the ages of 18–45 who were examined as part of a pre-employment physical. The subjects were assessed for PCOS according to the NIH guidelines. This study was detailed and avoided bias in the selection of their participants although, due to the chosen diagnostic criteria it did not include the polycystic ovarian morphology or ultrasound as part of the examination.

Other studies by Azziz et al. have examined this hypothesis using the same database and the same criteria [3]. These studies confirmed the finding of no significant difference, showing a prevalence of 8.0% for Black women and 4.8% for Caucasian women in Alabama [3]. The 2017 Chan et al. study comparing nationwide US white and black women initially showed a much higher prevalence for metabolic syndrome in US black women with PCOS, however, when adjusted for age and body mass index the prevalence actually turned out to be similar, denoting that even very recent research still indicates no significant difference between US black and white women [45].

#### 2.5.4. Indigenous Australian

Davis et al. reported results that are suggestive of a preliminary indication that indigenous Australian women could have a prevalence as high as 26%. It is important to note that this study is limited by its small sample size (*n* = 38) and a PCOS diagnosis based off of the presence of oligomenorrhea and hirsutism and/or hyperandrogenemia. The measures collected from the participants included hirsutism (from facial scoring only), BMI and waist circumference, insulin and glucose levels, and hormone analysis to test total testosterone and sex hormone binding globulin (SHBG), which were used to calculate a free androgen index [32]. It was expected that the prevalence found in the indigenous people would vary from the rest of the population, due to their rapid change from a hunter-gatherer way of life to a sedentary life-style with a high-fat and nutritionally poor diet. This population has especially high rates of hirsutism, central obesity, and type 2 diabetes compared to Caucasians [32]. These symptoms are thought to have an impact on the severity of the symptoms of PCOS and may be attributed to PCOS or increase their risk for PCOS.

#### 2.5.5. Greek Islanders

A cross-sectional study that assessed 192 women between the ages of 17 and 45 who were living on the Greek island of Lesbos determined the prevalence of PCOS, according to the NIH criteria to be 6.77% [46]. This study recruited participants via a convenience sample by accepting those who responded to their offer of a free medical examination by an endocrinologist. This method introduced bias and may have altered the results by attracting more individuals who think they need to see a doctor than those who consider themselves healthy. Regardless of the potential bias, this value parallels the typical prevalence rate in the United States [4]. Though few studies have been done specifically on Greek Island populations, a 2016 study done by Kyrkou et al. shows that metabolic syndrome does have quite a high prevalence in Greek women with PCOS at 12.6%, which is seven times higher than control populations [47] (see Table 4).

## 3. Conclusions

Based on the NIH diagnostic criteria, there is a similar prevalence of PCOS between 6% and 9% documented across the United States, the United Kingdom, Spain, Greece, Australia, Asia, and Mexico [48]. This information suggests that there are no racial or ethnic influences on the prevalence of PCOS. Due to the lack of comparability amongst the studies, biased group selections, and small sample sizes it is recommended that further research be conducted before this generalized statement is accepted. There are multiple hypothesized reasons for the lack of understanding of the risk and diagnosis of PCOS and one main reason could be the conflicting diagnostic criteria. The different components of the diagnostic criteria cause alterations in the prevalence across the NIH 1990 Criteria, Rotterdam 2003 Criteria, and AE-PCOS 2006 Criteria [6]. National prevalence rates have been reported as low as 1.6% using a combination of all three criteria [1] and as high as 6.6% using 1990 NIH criteria in similar American populations [3]. Due to the fact that PCOS only affects women of reproductive age, most of the studies looked at the age group between 18 and 45 [1,3,4,28,46]. While there is limited literature that exists, there have been similar prevalence rates between Caucasian people of European decent, African-American, and Mexican women noted [48]. Due to the inconsistency between diagnostic criteria and recruiting methods it is unlikely that all studies in this review are comparable enough to infer conclusive differences upon. The existing data is not conclusive enough to determine whether or not there is any significant differences in the prevalence of PCOS across geographical location, racial or ethnic groups.

### Future Research

Future research is needed to determine a better diagnostic criteria and ways to improve diagnosis so that less individuals with PCOS are undiagnosed. Conversely, improved diagnostic criteria will help reduce over diagnosis. These will be the first steps to determining a more accurate prevalence, which can then be assessed according to sub-populations to achieve a better understanding of this multifaceted syndrome. A better understanding will enhance clinical outcomes and patient benefit. This topic is in need of large-scale, random, population studies across the world that look at the prevalence of PCOS according to the established diagnostic criteria in specific sub-populations that can be repeated with many different sub-groups.

## Figures and Tables

**Table 1:** Diagnostic criteria for polycystic ovarian syndrome (PCOS).

National Institutes of Health (NIH) 1990 [17]	Rotterdam 2003 [7]	AE-PCOS Society 2006 [6]	NIH 2012/International PCOS Guidelines 2018 [14,15]
Hyperandrogenism Chronic Anovulation ---Both criteria needed	Hyperandrogenism Oligo-and/or anovulation Polycystic ovaries ---2 of 3 criteria needed	Hyperandrogenism Ovarian dysfunction ---Both criteria needed	Hyperandrogenism Oligo-and/or anovulation Polycystic ovaries ---2 of 3 criteria needed
First developed and most commonly used criteria today	Formulated to expand on NIH diagnostic definition	Formulated to provide an evidence-based definition	Encouraged a name change (2012 only) and identifying sub-phenotypes

**Table 2:** Summary of studies on PCOS prevalence in the United States.

Study Authors	Objectives	Main Findings	Strengths and Limitations
Okoroh et al. [1]	Compare regional prevalence of PCOS across US	47.5% prevalence in Southern Region 23.0% prevalence in North Central Region 18.7% prevalence in Western Region 10.3% in North Eastern Reion Across US population, 1.6% of women met at least one criterion for PCOS	Only study to assess US regional prevalence and include all available diagnostic criteria Prevalence estimate may be low due to retrospective design

**Table 3:** Summary of studies on prevalence in Caucasians.

Study Authors	Objectives	Main Findings	Strengths and Limitations
Asuncion et al. [28]	Assess prevalence in Caucasian women in Madrid, Spain	Incidence rate of 6.5% according to NIH criteria	Random selection to limit bias Small sample
March et al. [2]	Assess prevalence in Adelaide, Australia	8.7% prevalence by NIH criteria 11.9% by Rotterdam criteria 10.2% by AES criteria	Took into account all criteria Large community-based study

**Table 4:** Summary of studies on international prevalence.

Study Authors	Objectives	Main Findings	Strengths and Limitations
Kumarapeli et al. [37]	Assess prevalence in South Asian women ages 15–39	6.3% prevalence according to the Rotterdam criteria	Self-reported symptoms
Ding et al. [34]	Asses prevalence among Chinese, Caucasian, Middle Eastern, and African American	Chinese women had lowest prevalence at 5.6% according to Rotterdam criteria	
Chen et al. [42]	Assess prevalence among women in Southern China	2.2% prevalence according to NIH criteria	Possible selection bias
Moran et al. [43]	Assess prevalence in Mexico City, Mexico	6.0% prevalence by NIH criteria 6.6% prevalence by Rotterdam criteria	Used multiple criteria Small sample size
Goodarzi et al. [44]	Assess prevalence in Mexican-American women	Nearly 13% prevalence	Self-reported data Possible selection bias
Knochenhauer et al. [4]	Assess prevalence in Caucasian and Black women in Southeastern United States	4.7% prevalence in Caucasian women by NIH 3.4% prevalence in Black women by NIH	Minimal selection bias Diagnostic criteria did not include ultrasound or polycystic ovarian morphology
Azziz et al. [3]	Assess prevalence in Caucasian and Black women in Southeastern United States	8.0% prevalence in Black women according to NIH 4.8% prevalence in Caucasian women according to NIH	Minimal selection bias Diagnostic criteria did not include ultrasound or polycystic ovarian morphology
Davis et al. [33]	Assess prevalence in indigenous Australian women	Prevalence as high as 26% based off of presence of oligomenorrhea and hirsutism and/or hyperandrogenemia	Small sample size
Diamanti-Kandarakis et al. [46]	Assess prevalence in women on Greek island of Lesbos	6.6% prevalence according to NIH criteria	Convenience sample

## Abbreviations

PCOS	Polycystic Ovarian Syndrome
NIH	National Institute of Health
ESHRE	European Society of Human Reproduction and Embryology
ASRM	American Society for Reproductive Medicine
AE	Androgen Excess Society
ROT	Rotterdam criteria
US	United States of America
BMI	Body Mass Index
HOMA	Homeostasis Model Assessment
